# Time-course mass spectrometry data of adipose mesenchymal stem cells acquiring chondrogenic phenotype

**DOI:** 10.1186/s13104-018-3750-6

**Published:** 2018-09-12

**Authors:** Jeffrey R. Janus, Stephen G. Voss, Benjamin J. Madden, Mary Cristine Charlesworth, Michael S. Oldenburg, Dale Ekbom, Serban San-Marina

**Affiliations:** 1 Department of Otorhinolaryngology, Mayo Clinic, Rochester, MN USA; 2 Medical Genome Facility-Proteomics, Mayo Clinic, Rochester, MN USA; 3 Otolaryngology-Head and Neck Surgery, Prevea Health Services, Green Bay, WI USA

**Keywords:** Proteomics, Mass spectrometry, Adipose mesenchymal stem cells, Regenerative medicine, Chondrogenic differentiation time-course

## Abstract

**Objectives:**

Rabbit adipose mesenchymal stem cells were used for the purpose of studying acquisition of the chondrogenic phenotype over time at 1, 14 and 28 days after in vitro incubation with differentiation media, using nano-liquid chromatography electrospray ionization tandem mass spectrometry analysis. This was part of a preliminary study of the behavior of differentiated adipose stem cells for use in a rabbit model of laryngoplasty.

**Data description:**

The data comprise .MGF, .RAW, .MZID, and .XLSX, lists of peaks, peptides and proteins identified by nano-flow liquid chromatography electrospray ionization tandem mass spectrometry analysis upon incubation with non-differentiating (ND) or chondrogenic differentiating (CHD) media (ProteomeXchange ID PXD010236). XLSX files contain the following information: day 1 CT (control, N = 3499 proteins), day 14 ND (N = 3106 proteins), day 28 ND (N = 3116 proteins), day 14 CHD (N = 2901 proteins), and day 28 CHD (N = 2876 proteins). Proteins are characterized with respect to their − 10lgP value, percent coverage, number of total as well as unique peptides after trypsin digestion, derivatization method (carbamidomethylation, oxidation, or combined carbamidomethylation + oxidation), average mass, and include a full description.

## Objective

Adipose mesenchymal stem cells (ADSCs) are fast gaining popularity for various regenerative medicine applications due to their pluripotency as well as relative use of collection and processing. This has called for increased scrutiny and standardization of protocols involved in tissue collection, differentiation and analysis [1]. While analysis based on DNA or RNA expression has been the preferred method for studying gene expression due to the relatively low cost and flexible, scalable features of microarrays, these analyses do not report actual protein levels. Meanwhile, the cost of protein analysis using nano-flow liquid chromatography electrospray ionization tandem mass spectrometry (nLC–MS/MS) now allows for true protein measurements to be made at the tissue or even single cell level. Our lab’s interest lies in developing regenerative medicine application for otorhinolaryngology. Currently, we are gaining expertise with new biologicals to address vocal fold (VF) paralysis, one of the complications that can arise from accidental sectioning of the recurrent laryngeal nerve (RLN) during thyroidectomy. As part of developing a rabbit model for injection medialization laryngoplasty (IML), a surgical procedure involving injection of volume augmenting materials that bring the paralyzed VF proximal to its counterpart, we established protocols for growing and differentiating ADSCs into chondrocytes in vitro. The data now allow for time-course analyses of true protein levels with the potential of revealing new proteins and protein network models implicated in the chondrogenic differentiation of ADSCs in vitro.

## Data description

### Data description

Five lists of proteins were generated by nLC–MS/MS analysis of rabbit ADSC incubated with non-differentiating (ND) or chondrogenic differentiating (CHD) media for 1, 14, or 28 days. List 1: day 1 (N = 3499 proteins); List 2: ND media, day 14 (N = 3106 proteins); List 3: ND media, day 28 (N = 3116 proteins); List 4: CHD media, day 14 (N = 2901 proteins); and List 5: CHD media, day 28 (N = 2876 proteins). Proteins are displayed in rows and descriptors in columns, as follows: (1) Accession Number, (2) Protein Group, (3) Protein ID, (4) − 10 logP value, (5) % Coverage, (6) No. of Total Peptides, (7) No. of Unique Peptides, (8) Derivatization Method, (9) Average Mass, and (10) Description. A Protein Group is a list of proteins sharing a significant number of identical matched peptides. − 10 logP expresses the confidence of making a protein call; high values reflect high confidence [2]. Coverage refers to the percentage of amino acids in the matched peptides relative to the total number of amino acids in the protein.

### Chondrogenic differentiation

Chondrogenic differentiation was performed as previously described [3]. Briefly, ADSCs were prepared in 15 mL polystyrene tubes as collagen macrospheres by mixing 5 × 10^5^ cells with 50 µL of collagen followed by sedimentation at 500 g for 5 min and drop-wise addition of StemPro Chondrogenic differentiation media (Basal Medium + Chondrogenic Supplement, A10071-01 Life Technologies, Carlsbad, CA). This was supplemented with 5 ng/mL of TGF-β2 (T2815; Sigma-Aldrich, St. Louis, MO). Non-differentiating media consisted of the Basal Medium alone. All media was replenished every 2–3 days. Chondrogenic differentiation was confirmed by Alcian blue staining and sulfated glycosaminoglycan (s-GAG) level analysis with DNA content correction using PicoGreen (Quant-iT P7589, Invitrogen, Carlsbad, CA). Parallel cultures in adipogenic and osteogenic differentiation media (StemPro A1007001 and A1007201; ThermoFisher, Minneapolis, MN) were performed to confirm the presence of pluripotent stem cells in our samples. These data were published elsewhere [3].

### nLC–MS/MS analysis

After 1, 14, or 28 days the cell pellets were washed ten times with PBS, fixed in 4% paraformaldehyde and processed for nLc–MS/MS analysis as previously reported [4]. The sample preparation protocol is available on line (Table 1, File 21: Methods). Tandem MS/MS peptide spectra were extracted using msConvert software (ProteoWizard) and analyzed with Mascot (Matrix Science), and X! tandem in order to match mass spectra to peptide sequences. N-terminus ammonia loses (Glu → pyro-Glu and gln → pyro-Glu), oxidation of methionine and the iodoacetamide derivative of cysteine were specified as variable modifications A fragment ion mass tolerance of 0.020 Da and parent ion tolerance of 10.0 PPM were specified in Mascot and X! Tandem. Scaffold (version 4.6.2, from Proteome Software Inc., Portland, OR) was used to validate MS/MS based peptide and proteins. The Rabbit RefSeq database (12/2016 with 77,162 entries) was searched and hits verified against a decoy database. Peptide IDs were accepted if they could be established at greater than 95% probability by the Scaffold Local FDR algorithm. Protein IDs were accepted if they could be established at greater than 95% probability using Protein Prophet [5]. Only proteins containing two or more unique peptides were compared across groups [6].

**Table 1:** Overview of data files/data sets

Label	Name of data set	File types (file extension)	Data repository and identifier (DOI or accession number)
File 1	Day 1 Ctrl	MS/MS spectra file (.mgf)	ftp://massive.ucsd.edu/MSV000082505/peak/day1ctrl.mgf
File 2	Day 1 Ctrl	Mass spectrometry data (.raw)	ftp://massive.ucsd.edu/MSV000082505/raw/day1ctrl.raw
File 3	Day 1 Ctrl	Peptide data (.mzid)	ftp://massive.ucsd.edu/MSV000082505/result/day1ctrl_peptides_1_1_0.mzid
File 4	ND day 14	MS/MS spectra file (.mgf)	ftp://massive.ucsd.edu/MSV000082505/peak/day14ctrl.mgf
File 5	ND day 14	Mass spectrometry data (.raw)	ftp://massive.ucsd.edu/MSV000082505/raw/day14ctrl.raw
File 6	ND day 14	Peptide data (.mzid)	ftp://massive.ucsd.edu/MSV000082505/result/day14ctrl_peptides_1_1_0.mzid
File 7	ND day 28	MS/MS spectra file (.mgf)	ftp://massive.ucsd.edu/MSV000082505/peak/day28ctrl.mgf
File 8	ND day 28	Mass spectrometry data (.raw)	ftp://massive.ucsd.edu/MSV000082505/raw/day28ctrl.raw
File 9	ND day 28	Peptide data (.mzid)	ftp://massive.ucsd.edu/MSV000082505/result/day28ctrl_peptides_1_1_0.mzid
File 10	CHD day 14	MS/MS spectra file (.mgf)	ftp://massive.ucsd.edu/MSV000082505/peak/day14ctrl.mgf
File 11	CHD day 14	Mass spectrometry data (.raw)	ftp://massive.ucsd.edu/MSV000082505/raw/day14chond.raw
File 12	CHD day 14	Peptide data (.mzid)	ftp://massive.ucsd.edu/MSV000082505/result/day14chond_peptides_1_1_0.mzid
File 13	CHD day 28	MS/MS spectra file (.mgf)	ftp://massive.ucsd.edu/MSV000082505/peak/day28chond.mgf
File 14	CHD day 28	Mass spectrometry data (.raw)	ftp://massive.ucsd.edu/MSV000082505/raw/day28chond.raw
File 15	CHD day 28	Peptide data (.mzid)	ftp://massive.ucsd.edu/MSV000082505/result/day28chond_peptides_1_1_0.mzid
File 16	Day 1 Ctrl	Protein list file (.xlsx)	ftp://massive.ucsd.edu/MSV000082505/updates/2018-07-19_ssanmarina1_eec23ce3/other/Day1_ctrl_proteins.xlsx
File 17	ND day 14	Protein list file (.xlsx)	ftp://massive.ucsd.edu/MSV000082505/updates/2018-07-19_ssanmarina1_eec23ce3/other/Day14_ctrl_proteins.xlsx
File 18	CHD day 14	Protein list file (.xlsx)	ftp://massive.ucsd.edu/MSV000082505/updates/2018-07-19_ssanmarina1_eec23ce3/other/Day14_chondro_proteins.xlsx
File 19	ND day 28	Protein list file (.xlsx)	ftp://massive.ucsd.edu/MSV000082505/updates/2018-07-19_ssanmarina1_eec23ce3/other/Day28_ctrl_proteins.xlsx
File 20	CHD day 28	Protein list file (.xlsx)	ftp://massive.ucsd.edu/MSV000082505/updates/2018-07-19_ssanmarina1_eec23ce3/other/Day28_chondro_proteins.xlsx
File 21	Methods	Methods file (.docx)	ftp://massive.ucsd.edu/MSV000082505/methods/ProteomeXchange%20project%20description_final.docx

## Limitations

The data presented here describe acquisition of the chondrogenic phenotype by rabbit ADSCs. The data are useful for the appreciation of time-dependent cellular changes in ADSCs at 14 and 28 days of incubation with chondrogenic differentiation media. The data are also useful for a comparison across species, for example with mouse or human ADSCs, aimed at identifying patterns of similar or differential expression of proteins implicate in acquisition of the chondrogenic phenotype. The main limitations of the data are the lack of replicate measurements and time points beyond day 28. Furthermore, because of the lack of a finer temporal resolution, transient, or intermediary events are not properly captured.

## Abbreviations

ADSC: adipose mesenchymal stem cells
CHD: chondrogenic differentiating (media)
IML: injection medialization laryngoplasty
ND: non-differentiating (media)
nLc–MS/MS: nano-liquid chromatography electrospray ionization tandem mass spectrometry
RLN: recurrent laryngeal nerve
s-GAG: sulfated glycosaminoglycan
VF: vocal fold
